# Integrative Transcriptomic and Network Pharmacology Analysis Reveals Key Targets and Mechanisms of *Moschus* (musk) Against Viral Respiratory Tract Infections

**DOI:** 10.3390/ph18081136

**Published:** 2025-07-30

**Authors:** Ke Tao, Li Shao, Haojing Chang, Xiangjun Chen, Hui Xia, Ruipeng Wu, Shaokang Wang, Hehe Liao

**Affiliations:** 1 Key Laboratory of Environmental Medicine and Engineering of Ministry of Education, Department of Nutrition and Food Hygiene, School of Public Health, Southeast University, Nanjing 210009, China; augpeche20@163.com (K.T.); shaoli@xzmu.edu.cn (L.S.); chj@seu.edu.cn (H.C.); xjchen@xzmu.edu.cn (X.C.); huixia@seu.edu.cn (H.X.); 2 Clinical Medical Research Center for Plateau Gastroenterological Disease of Xizang Autonomous Region, School of Medicine, Xizang Minzu University, Xianyang 712082, China; wurp@xzmu.edu.cn

**Keywords:** *Moschus*, viral respiratory tract infections, network pharmacology, multi-omics integration, molecular docking, structural biotechnology

## Abstract

**Background/Objectives**: *Moschus* (musk) has long been used in traditional Tibetan medicine to prevent and treat epidemic febrile illnesses. However, its antiviral mechanisms remain poorly understood. Given the urgent need for effective treatments against viral respiratory tract infections (VRTIs), this study aimed to systematically investigate the molecular targets and pharmacological pathways through which Moschus may exert therapeutic effects. **Methods**: Based on the identification of bioactive compounds with favorable pharmacokinetics, we applied integrated network pharmacology and multi-omics analyses to systematically identify key therapeutic targets involved in VRTIs. Gene Set Enrichment Analysis (GSEA) and immune infiltration further revealed strong associations with multiple immune cell subsets, reflecting their pivotal roles in immunomodulatory mechanisms during viral infections. Molecular docking confirmed the strong binding affinities between *Moschus* compounds and these key targets. **Results**: Notably, testosterone exhibited the strongest and most consistent binding across key targets, suggesting its potential as a pivotal bioactive compound. Importantly, the antiviral effects of *Moschus* may be mediated in part by the downregulation of the key genes MCL1, MAPK3, and CDK2, which are involved in the regulation of viral replication, apoptosis, and host immune responses. **Conclusions**: This study provides a comprehensive mechanistic framework supporting the multi-target antiviral potential of *Moschus*, offering a scientific basis for its further development as a therapeutic agent against VRTIs.

## 1. Introduction

Viral respiratory tract infections (VRTIs) are respiratory diseases caused by a variety of viruses, ranging from mild illnesses such as the common cold to more severe conditions including pneumonia and acute respiratory distress syndrome (ARDS). Common etiological agents include rhinoviruses, influenza viruses, respiratory syncytial virus, coronaviruses, adenoviruses, and others [1].Viruses infect the respiratory epithelium primarily through droplet and direct contact transmission, initiating replication and inducing the production of interferons as well as the release of pro-inflammatory cytokines [2,3]. These processes promote immune cell infiltration and activate adaptive immune responses. However, a dysregulated immune response may lead to airway injury and secondary infections [4,5], potentially resulting in respiratory dysfunction or even death in severe cases [6,7]. With increasing global population mobility and the continual emergence of novel pathogens, VRTIs have become a major threat to global public health. Although vaccines and antiviral agents have been developed for the prevention or treatment of certain viral infections, the high diversity of pathogens, antigenic drift, and the challenges posed by an aging population continue to exert significant pressure on effective disease control.

Traditional Tibetan medicine is an integral component of traditional Chinese medicine (TCM), characterized by a long-standing history and extensive empirical knowledge. In Tibetan medical theory, the human body is viewed as a dynamic and holistic system, with the onset of disease closely associated with imbalances among the three fundamental physiological factors—Loong, Tripa, and Beken—as well as external influences such as cold, heat, and dampness [8]. When exogenous pathogenic factors invade the lungs or airways, they may disrupt the equilibrium of these three factors, leading to respiratory symptoms such as fever, cough, and dyspnea—hallmarks of respiratory tract infections. Modern pharmacological studies have demonstrated that many Tibetan medicines possess significant anti-inflammatory, antiviral, immunomodulatory, and antioxidant activities. These effects are believed to be mediated through the regulation of multiple molecular targets. In contrast, current pharmacological treatments for viral respiratory tract infections often face limitations, including suboptimal efficacy, notable side effects [9,10], the emergence of drug resistance [11], and restricted applicability across different patient populations. Owing to their multi-target mechanisms, low toxicity, and holistic regulatory properties, Tibetan medicines and other natural products have shown promising therapeutic potential in the management of VRTIs.

*Moschus* (musk) is a dried secretion derived from the musk gland of adult male musk deer, as documented in *Chinese Tibetan Materia Medica*. In traditional Tibetan medicine, *Moschus* is a commonly used remedy for treating epidemic febrile diseases classified as *gnyan rims*, which encompass conditions associated with plagues and febrile diseases [12,13]. According to the *Jingzhu Materia Medica*, *Moschus* possesses properties such as detoxification, insecticidal activity, anti-inflammation, and the ability to ward off pathogenic influences [14]. Notably, *Moschus* serves as the principal ingredient in the classical Tibetan formulation “Nine musk pill (Jiuwei shexiangwan)”, which has been employed both orally and as an aromatic agent to prevent and alleviate symptoms of infectious disease [15].

Phytochemical and modern pharmacological studies have revealed that *Moschus* contains various bioactive compounds, including macrocyclic ketones, pyridines, steroids, fatty acids, amino acids, peptides, and proteins. Among these, muscone, a macrocyclic ketone, is recognized as the principal active compound and has demonstrated anti-inflammatory, neuroprotective, antitumor, and cardioprotective effects in experimental studies [16,17]. These findings provide both theoretical and empirical support for the antimicrobial and anti-inflammatory properties of *Moschus*. However, no systematic investigation has yet been conducted to elucidate the specific roles and underlying mechanisms of its active constituents in the prevention or treatment of respiratory tract infections. Therefore, further comprehensive studies are warranted to clarify their therapeutic potential.

Natural compounds, particularly bioactive constituents derived from traditional Chinese medicine, have demonstrated unique advantages in modulating immune responses and exerting antiviral effects, making them a growing focus of contemporary biomedical research. In this context, it is imperative to identify key regulatory factors and immune cell interaction networks involved in viral infections through integrative approaches such as multi-omics analyses and systems biology. These approaches are essential for uncovering novel therapeutic targets. This study aims to systematically elucidate the potential mechanisms of *Moschus* in treating VRTIs by integrating gene expression data from the GEO database with network pharmacology approaches. The findings are expected to provide a solid theoretical foundation for the development of novel antiviral therapeutics.

## 2. Results

### 2.1. Screening of Active Compounds and Potential Targets

After literature retrieval and the exclusion of compounds that did not meet the screening criteria, a total of 21 candidate compounds were retained. Target prediction was subsequently conducted for these compounds. Compounds without predicted targets, unmatched genes, and duplicate targets were excluded. Ultimately, 12 bioactive compounds and 196 target genes were identified (Table 1).

### 2.2. Retrieval Results of VRTI Targets

Target genes associated with VRTIs were retrieved from the GeneCards database using the keyword “viral respiratory tract infections” and a relevance score threshold of ≥10. Additional targets were retrieved from the TTD, OMIM, and PharmGKB databases. After merging the results from all databases and removing duplicates, a total of 2875 VRTI-related targets were obtained.

### 2.3. Screening of Core Drug–Disease Targets Based on PPI Network

**Figure 1:**
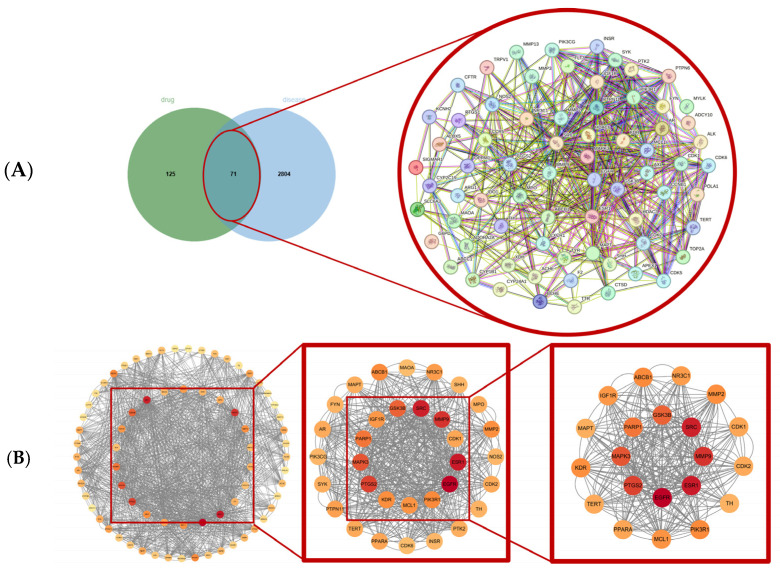
Screening of core drug–disease targets based on PPI network. (**A**) Identification and visualization of overlapping targets between *Moschus* and VRTI-related genes. (**B**) Workflow of core gene identification from the PPI network of *Moschus*–VRTI overlapping targets.

A Venn diagram was constructed to identify the intersection between 196 drug-related targets and 2875 VRTI-related disease genes, resulting in 71 overlapping targets considered as potential therapeutic candidates. These common targets were imported into the STRING database to analyze PPI information (Figure 1A). In the resulting network, nodes represent proteins, and edges represent the interactions between proteins. The PPI network was then imported into Cytoscape 3.10.3 to construct a visualization network, where the size and color gradient of each node are adjusted based on the degree value. Darker colors and larger nodes indicate higher degree values, suggesting greater functional importance and more complex mechanisms of action. The *Moschus*–VRTI network comprised 71 nodes and 547 edges, with a highly significant PPI enrichment *p*-value < 1.0 × 10^−16^. Using the CytoNCA plugin, six topological parameters—BC, CC, DC, EC, LAC, and NC—were calculated. Targets with values above the median degree for all parameters were selected, resulting in the identification of 21 hub genes (Figure 1B, Table 2).

### 2.4. GEO Data Collection and DEG Analysis

To investigate host transcriptomic responses to viral infections, three publicly available gene expression datasets were retrieved from the GEO database. The included datasets comprised both uninfected controls and infected individuals, with sample types collected from whole blood or peripheral blood mononuclear cells (PBMCs). Detailed dataset characteristics are summarized in Table 3.

**Figure 2:**
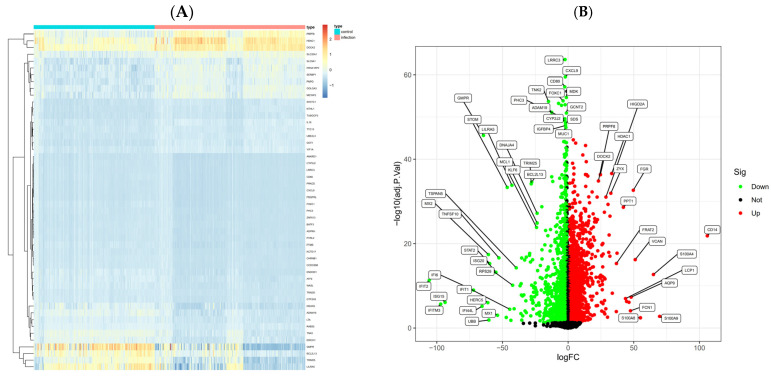
Differentially expressed genes after batch effect correction. (**A**) Volcano plot showing 1221 upregulated (red) and 820 downregulated (green) genes. (**B**) Heatmap displaying expression profiles of the DEGs across samples.

**Figure 3:**
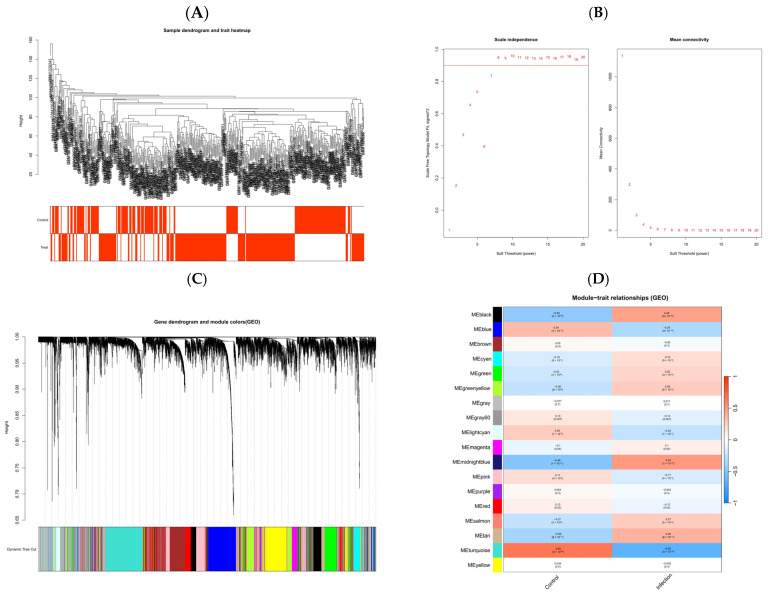
WGCNA. (**A**) Sample dendrogram and trait heatmap. (**B**) Analysis of soft threshold power showing scale independence and mean connectivity. Numbers indicate soft-thresholding powers tested; curves show scale independence and mean connectivity for each power to help select the optimal threshold. (**C**) Gene clustering dendrogram and module colors. (**D**) Module–trait heatmap.

After batch effect removal and differential expression analysis on the three datasets [18], a total of 2041 differentially expressed genes (DEGs) were identified. The volcano plot displays these results, with red dots representing 1221 upregulated genes and green dots representing 820 downregulated genes (Figure 2A,B), alongside the corresponding heatmap. Subsequently, the gene expression matrix was subjected to WGCNA (Figure 3A–D). Sample clustering showed even grouping without significant outliers. Scale-free topology analysis determined the optimal soft threshold to be 7. Dynamic tree cut analysis identified 18 gene modules, as shown in the module–trait heatmap, revealing significant associations between key modules and viral respiratory tract infection status. Notably, the MEturquoise module, which contains 1532 genes, was strongly positively correlated with infection status, with a correlation coefficient =0.65 and a *p* of 3 × 10^−53^. Genes from modules with *p* < 0.05 in the module–trait heatmap were extracted and deduplicated for subsequent key gene screening.

### 2.5. Screening of Key Genes

**Figure 4:**
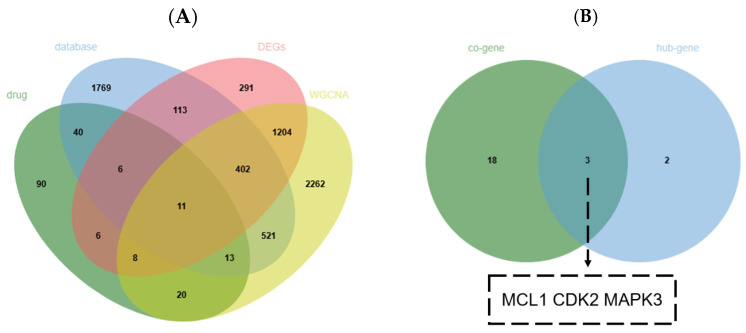
Venn diagrams of core genes screening in VRTIs. (**A**) Intersection of drug targets, disease genes, and transcriptomic data. (**B**) Further screening of key genes by intersecting PPI core genes with hub genes.

To identify pivotal genes implicated in VRTIs, we integrated drug targets, disease-associated genes, DEGs, and WGCNA modules with significant phenotypic associations. Venn diagram analysis revealed 11 hub genes (Figure 4A) [19]. Subsequently, we intersected these hub genes with the 21 core genes previously identified through PPI network topological analysis, refining the candidate list to three key genes—MCL1, CDK2, and MAPK3—that showed robust associations with disease pathogenesis (Figure 4B).

### 2.6. Gene Ontology and KEGG Pathway Analysis

**Figure 5:**
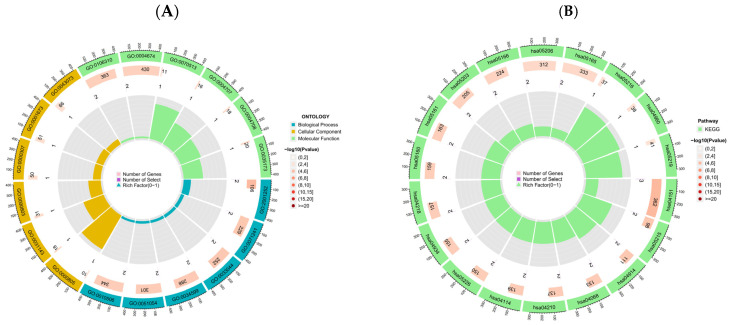
(**A**) Gene Ontology (GO) enrichment Circos plot of the identified key genes. (**B**) KEGG pathway enrichment Circos plot of the identified key genes.

As depicted in Figure 5A, in the biological process category, genes were enriched in the positive regulation of chromosome organization, cellular response to inorganic substance, regulation of chromosome organization, cellular response to oxidative stress, positive regulation of DNA metabolic process, and regulation of autophagy. For cellular components, enrichment was observed in cell structures associated with viral infection and host–pathogen interactions, including the plasma membrane raft, membrane raft, membrane microdomain, mitochondrial outer membrane, outer membrane, endoplasmic reticulum lumen, and late endosome. Molecular functions analysis indicated enrichment in protein kinase activity, including protein serine/threonine kinase activity, MAP kinase activity, histone kinase activity, and cyclin-dependent protein kinase activity.

Molecular functions analysis indicated that protein kinase activity, including protein serine/threonine kinase activity, MAP kinase activity, histone kinase activity, and cyclin-dependent protein kinase activity, were among the most enriched terms.

The KEGG pathway analysis revealed significant pathways associated with the PI3K-Akt signaling pathway, apoptosis, the Toll-like receptor signaling pathway, the FoxO signaling pathway, viral carcinogenesis, human T-cell leukemia virus 1 infection, human papillomavirus infection, and the p53 signaling pathway (Figure 5B).

### 2.7. Single-Gene Gene Set Enrichment Analysis

**Figure 6:**
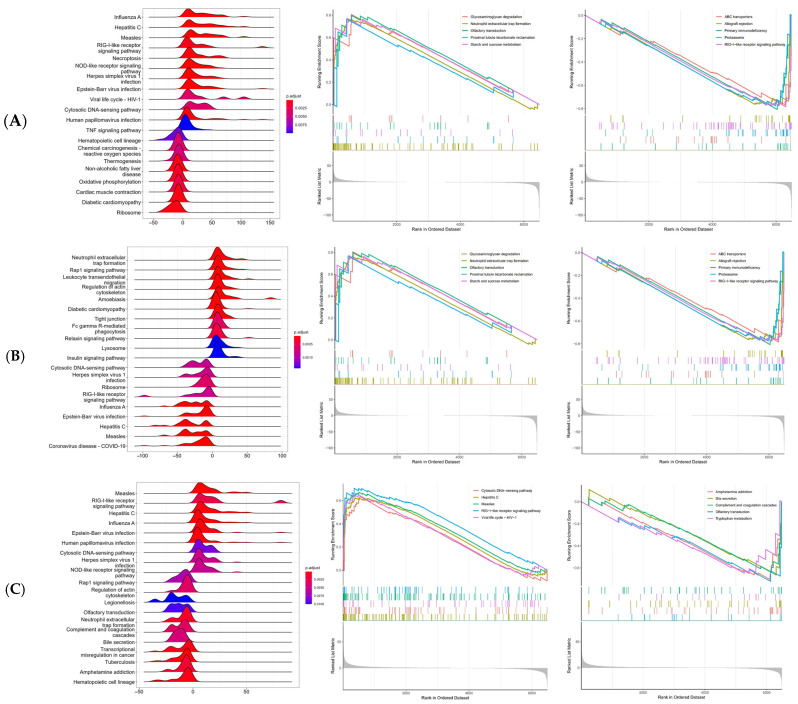
Ridge plots and running enrichment curves for the top five positively and negatively enriched pathways of (**A**) MCL1, (**B**) MAPK3, and (**C**) CDK2.

Using GEO transcriptomic data, we performed single-gene Gene Set Enrichment Analysis (GSEA) on MCL1, MAPK3, and CDK2 to explore their potential functions and regulatory roles in VRTIs. As shown in the ridge plots (Figure 6A–C), each key gene exhibited distinct enrichment profiles across various functional pathways. Specifically, MCL1 was significantly enriched in pathways related to viral infection, including hepatitis C, influenza A, measles, the RIG-I-like receptor signaling pathway, and the HIV-1 viral life cycle pathway. MAPK3 showed significant enrichment in the Rap1 signaling pathway, the regulation of actin cytoskeleton, influenza A, and COVID-19 pathways. CDK2 was significantly enriched in multiple immune- and virus-related pathways, including the Rap1 signaling pathway, regulation of actin cytoskeleton, RIG-I-like receptor signaling, NOD-like receptor signaling, influenza A, and hepatitis C. To visualize the distribution of significantly enriched pathways, the top five upregulated and downregulated gene sets were selected based on their enrichment scores. Enrichment plots were then generated to show the differences in enrichment trends between high- and low-expression groups.

### 2.8. Immune Response Signatures and Associations with Key Genes

**Figure 7:**
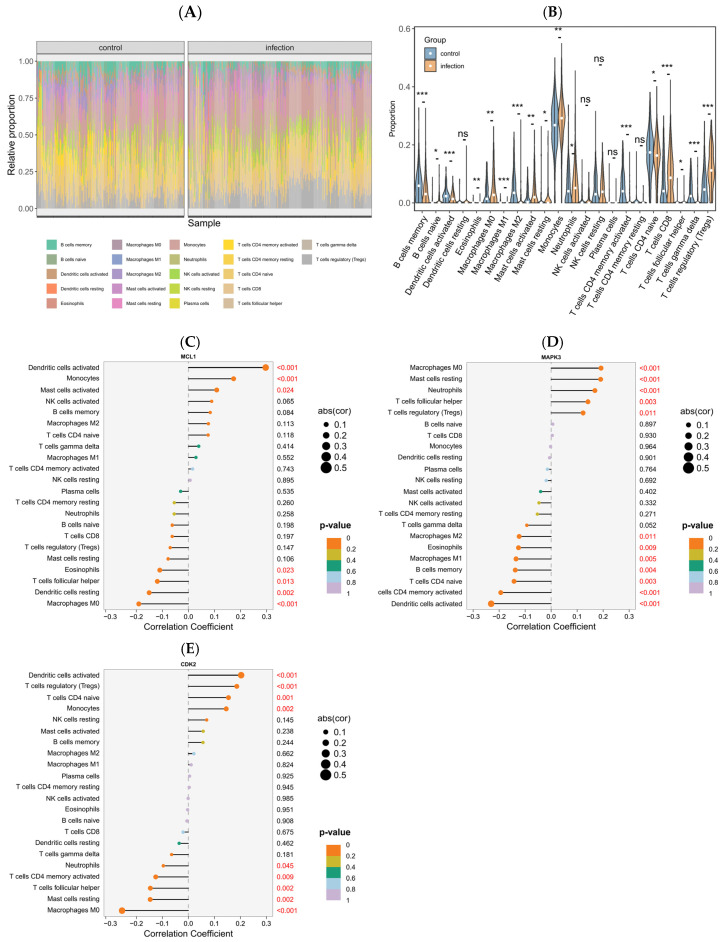
Immune infiltration analysis of core genes. (**A**) Relative proportions of 22 immune cell subsets in control and infected samples. (**B**) Comparison of immune cell proportions between groups. (**C**–**E**) Correlation of MCL1, MAPK3, and CDK2 expression with immune cell infiltration, respectively. The boxplots represent the interquartile range and median, with white dots indicating the group-wise median values. Statistical significance was determined using the Wilcoxon rank-sum test; *p*-values are denoted as *p* ≤ 0.001 (***), *p* ≤ 0.01 (**), *p* ≤ 0.05 (*), and *p* > 0.05 (ns). Groups are color-coded as indicated.

As illustrated in violin and box plots (Figure 7A,B), the distribution of immune cell populations differed significantly between the control and infection groups. A marked increase in the infiltration of several immune cell subsets, including CD8+ T cells, regulatory T cells (Tregs), M0 macrophages, activated mast cells, and monocytes, was observed in the infection group compared to the control group. Conversely, the proportions of memory B cells, activated dendritic cells, γδ T cells, M1 macrophages, and M2 macrophages were significantly reduced in the infection group.

To further explore the immunogenomic interactions, we performed Spearman correlation analysis between the expression of key genes and infiltration levels of various immune cells. Results were visualized as lollipop plots, where line length represents the correlation coefficient, and dot color reflects statistical significance, with orange representing higher significance (lower *p*-values), and purple representing lower or non-significant associations. As shown in Figure 7C–E, MCL1 expression was significantly positively correlated with several immune cell subsets, including activated dendritic cells, monocytes, and activated mast cells. In contrast, eosinophils, follicular helper T cells, resting dendritic cells, and M0 macrophages exhibited negative correlations with MCL1 expression. MAPK3 expression displayed strong positive associations with M0 macrophages, resting mast cells, neutrophils, and follicular helper T cells, while showing negative correlations with activated dendritic cells, activated memory CD4+ T cells, naive CD4+ T cells, and memory B cells. CDK2 expression was positively correlated with activated dendritic cells, Tregs, naive CD4+ T cells, and monocytes. In contrast, negative correlations were observed between CDK2 expression and M0 macrophages, resting mast cells, follicular helper T cells, and activated memory CD4+ T cells.

### 2.9. Molecular Docking Validation Results

**Figure 8:**
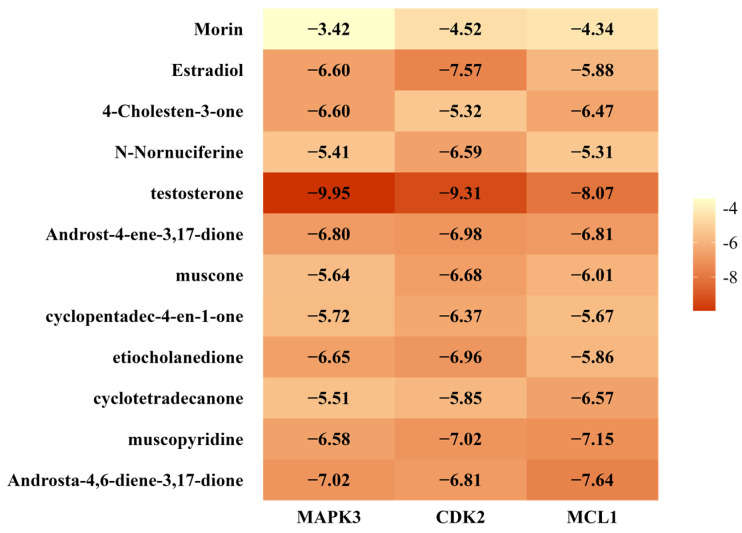
Heatmap of molecular docking scores between main compounds and key targets.

**Figure 9:**
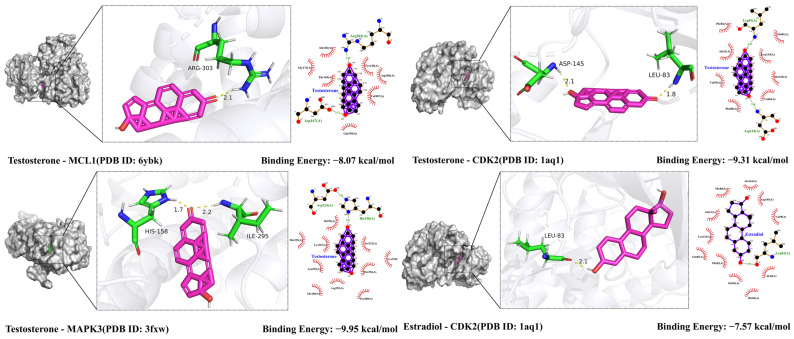

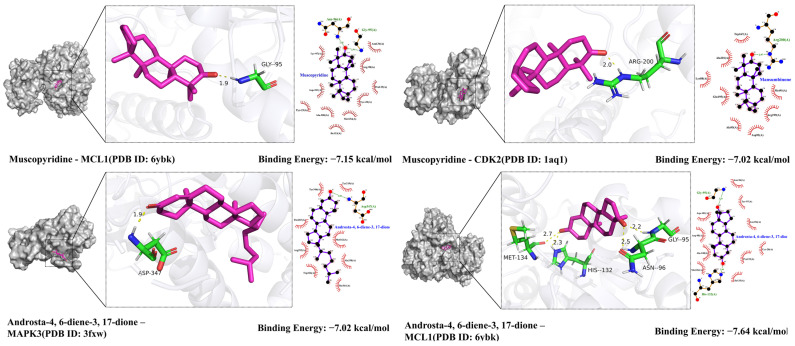
Three- and two-dimensional interaction diagrams of molecular docking between main compounds and key targets (MCL1, MAPK3, CDK2) with binding energies <−7 kcal/mol.

The key target proteins MCL1 (PDB ID: 6YBK), MAPK3 (PDB ID: 3FXW), and CDK2 (PDB ID: 1AQ1) were selected as macromolecular targets, and 12 active compounds from *Moschus* were used as ligands for molecular docking analysis. The binding free energy of each target is presented in Figure 8. Except for Morin, the remaining 11 active compounds exhibited binding energies below −5 kcal/mol, indicating strong binding affinities. The strongest binding affinity was observed between testosterone and MAPK3 (–9.95 kcal/mol). Additionally, the top eight ligand–macromolecular target pairs with the lowest binding free energies were visualized to show their molecular interactions (Figure 9).

## 3. Discussion

VRTIs are typically transmitted via respiratory droplets or aerosols. Upon entering the respiratory tract, viruses invade host cells by binding to specific surface receptors, primarily targeting airway epithelial cells [20]. Viral infection induces the expression of type I and type III interferons, along with various pro-inflammatory cytokines and chemokines, leading to immune dysregulation. This imbalance may trigger a cytokine storm, tissue damage, and disruption of the epithelial barrier. Mild cases present with respiratory symptoms, whereas severe cases can progress to pneumonia, secondary infections, sepsis, multiple organ failure, and even death. The high incidence and mortality rates of VRTIs impose a substantial disease burden and place significant strain on healthcare systems worldwide [21]. Current antiviral strategies include direct-acting antiviral drugs [22,23], host-directed antivirals [24,25,26,27], monoclonal antibodies, and protease inhibitors [28]. However, these therapies face challenges such as drug resistance [29,30], adverse effects [10,31,32], and a narrow antiviral spectrum [33]. In response to these challenges, *Moschus*, a traditional Tibetan medicine, has emerged as a promising candidate. Its multi-target and multi-pathway pharmacological characteristics offer the potential to overcome the limitations of conventional antiviral therapies.

The present study systematically integrated data from the literature and multiple databases to screen and identify 12 active compounds in *Moschus* and their corresponding 196 potential target genes. Among the 12 candidate compounds identified, several have been extensively investigated and shown to exhibit diverse pharmacodynamic properties pertinent to the pathogenesis of VRTIs. Morin has been reported to exhibit broad-spectrum antiviral activity and may alleviate VRTIs by suppressing pro-inflammatory cytokine production and reducing oxidative stress [34,35] and inhibiting viral replication [36]. These effects are primarily mediated through the inhibition of the MAPK and NF-κB pathways [37,38], as well as the activation of the Nrf2/HO-1 signaling pathway. Testosterone suppresses excessive immune responses by modulating androgen receptor signaling and maintaining T-cell homeostasis [39,40]. It also attenuates pulmonary inflammation by downregulating pro-inflammatory cytokines such as IL-1β, IL-6, and TNF-α [41,42]. Furthermore, in a rat model of chronic obstructive pulmonary disease, testosterone supplementation has been shown to inhibit the activation of NRF1 and p65 in lung epithelial cells, thereby reducing pulmonary inflammatory responses [43]. Estradiol, which is derived from peripheral testosterone conversion, signals through ERα to dampen inflammation and improve outcomes following influenza A virus infection in female mice [44]. Specifically, estradiol alleviates virus-induced immunopathology by downregulating the expression of pro-inflammatory cytokines such as TNF-α, IL-6, and CCL2, thereby mitigating excessive pulmonary inflammation and improving infection outcomes [45,46]. Recent studies on muscone have primarily focused on its anti-inflammatory mechanisms, demonstrating that it suppresses the expression of pro-inflammatory cytokines such as IL-6, IL-1β, and TNF-α [47,48], modulates inflammation-related signaling pathways [16,49], and consequently alleviates immune–inflammatory responses. Evidence from a cross-sectional study [50] indicates that Androst-4-ene-3,17-dione, as the primary precursor of testosterone, contributes to elevated testosterone levels, which in turn enhance the hormone’s inhibitory effect on inflammation. The remaining active compounds in *Moschus* currently lack documented pharmacological evidence regarding their pharmacological roles in VRTIs or related mechanisms and therefore require further investigation.

Through intersection analysis with 2875 VRTI-related targets obtained from four disease databases, 71 common targets were identified. Subsequently, a PPI network was constructed based on the STRING database, and topological analysis was performed to screen 21 potential gene targets. Considering that the PPI network does not reflect expression activity under disease conditions, transcriptome data from GEO were further incorporated for biological validation through DEGs and WGCNA. The integration of three datasets yielded a total of 2041 DEGs and 18 co-expression modules. By intersecting DEGs, key genes from significantly associated modules, drug targets, and disease targets—MCL1, MAPK3, and CDK2—were ultimately identified.

KEGG and GO enrichment analyses revealed that core genes were significantly involved in multiple canonical immune- and apoptosis-related pathways, including the PI3K-Akt signaling pathway [51,52], apoptosis, Toll-like receptor signaling, the FoxO signaling pathway, and the p53 signaling pathway, as well as virus-associated pathways such as viral carcinogenesis, HTLV-1 infection, and human papillomavirus infection. These pathways are closely associated with host immune responses, cell survival, and inflammation during viral infection [53,54,55,56].

Molecular docking further supported the functional relevance of the key targets identified in this study and provided additional structural insights into the potential mechanisms by which Moschus may exert its therapeutic effects against VRTIs. The docking results revealed that 11 out of the 12 active compounds, with the exception of morin, exhibited binding free energies below −5 kcal/mol, indicating strong binding affinities with their respective target proteins. Among the Moschus-derived compounds screened in this study, testosterone demonstrated the strongest binding affinities toward key targets associated with VRTIs, including MAPK3, CDK2, and MCL1 (with binding energies < −8 kcal/mol). This finding, which is supported by existing clinical evidence, highlights testosterone as a potentially relevant bioactive compound worthy of further investigation.

Recent studies have shown that testosterone can modulate immune responses by enhancing FOXP3 expression, promoting regulatory T-cell differentiation, and suppressing the production of pro-inflammatory cytokines [57]. Notably, low circulating testosterone levels have been associated with increased inflammatory markers and worse clinical outcomes in male COVID-19 patients, suggesting a mechanistic link between androgen signaling and immune dysregulation during viral infection [58,59]. Moreover, several studies have demonstrated that exogenous testosterone supplementation can inhibit key pro-inflammatory cytokines, such as IL-1, IL-6, and TNF-α, thereby alleviating the inflammation associated with low endogenous testosterone levels [42,60,61]. Notably, unlike corticosteroids, testosterone may attenuate excessive inflammatory responses without compromising antiviral immune defense, suggesting a potential therapeutic advantage in the context of viral infections [41]. Although these findings provide biologically plausible hypotheses, they are primarily based on computational predictions and thus warrant further experimental validation. The dosage requirements, sex-specific effects, and safety profile of testosterone in this context remain unclear. Given its systemic endocrine functions, additional mechanistic and in vivo studies are necessary to elucidate testosterone’s potential therapeutic relevance and precise antiviral mechanisms within Moschus-based interventions.

MCL1, a key anti-apoptotic member of the Bcl-2 family, plays a dual role during viral infections. It has been reported that MCL1 functions as a key host factor that restricts avian coronavirus infectious bronchitis virus (IBV) infection replication and spread by modulating virus-induced apoptosis [62]. In SARS-CoV-2 infection, MCL1 is exploited by SARS-CoV-2 N protein to suppress apoptosis and enhance viral replication [63]. These findings suggest that MCL1 may function as a pathogen-specific molecular switch, modulating the balance between viral clearance and survival [64]. Previous studies have demonstrated that androgen deprivation suppresses androgen receptor (AR) signaling, resulting in decreased or dysregulated MCL1 expression and increased apoptosis in prostate cancer cells [65]. Based on these observations, we hypothesize that testosterone may modulate MCL1 expression via AR-mediated cell cycle pathways, thereby influencing host antiviral responses. GSEA further revealed that MCL1 is significantly enriched in multiple virus-related pathways, including hepatitis C, influenza A, measles, the RIG-I-like receptor signaling pathway, and the HIV-1 viral life cycle. This enrichment profile is consistent with the recognized functions of MCL1 and suggests that it may contribute to viral clearance by influencing both cell death and homeostatic mechanisms [66,67]. Immune infiltration analysis supports these findings by demonstrating that MCL1 expression is positively correlated with activated dendritic cells, monocytes, and activated mast cells, and negatively correlated with eosinophils, follicular helper T cells, and M0 macrophages. This indicates that MCL1 may contribute to immune cell polarization and recruitment within the inflammatory microenvironment of virus-induced lung injury [68]. KEGG and GO enrichment analyses further confirm that MCL1 predominantly operates through the PI3K-Akt-GSK-3 axis and Toll-like receptor signaling to regulate apoptosis and maintain immune homeostasis during viral infection.

Current evidence supports MCL1 as a promising antiviral target, but its regulation by testosterone remains largely inferred from non-infectious models like prostate cancer. Whether similar AR–MCL1 signaling operates in virus-infected airway cells remains unknown. Future in vitro and in vivo studies are needed to further validate the efficacy of this regulatory mechanism.

Mitogen-activated protein kinase 3 (MAPK3), which is also known as extracellular signal-regulated kinase 1 (ERK1), is a key component of the ERK/MAPK signaling cascade, which regulates a range of cellular processes [69]. Previous studies have demonstrated that MAPK3 facilitates the S-phase entry of host cells during HSV-1 infection by activating cyclin E and CDK2, thereby creating a cellular environment favorable for viral replication [70]. Additionally, MAPK3 modulates the immune landscape by regulating T-cell apoptosis and maintaining the balance between Tregs and T-helper 17 cells via the ERK1/ETS2/AURKA/NF-κB/Fas axis [71]. GSEA analysis indicated that MAPK3 is significantly enriched in antiviral pathways, including those related to influenza, hepatitis B, and measles viruses, suggesting its pivotal role in the host antiviral defense. Moreover, immune infiltration analysis showed increased levels of M0 macrophages, CD8+T cells, and Tregs in virus-infected samples, alongside decreased memory B cells and M2 macrophages, indicating an immune imbalance associated with infection [72]. Additionally, KEGG and GO enrichment analyses further supported these findings by demonstrating significant enrichment of MAPK3 in signaling pathways related to influenza, hepatitis B, and measles viruses. Existing evidence indicates that CDK2 phosphorylates viral proteins such as SARS-CoV-2 nsp12 and HIV Tat, thereby enhancing viral RNA synthesis and transcriptional activity [73,74]. In addition, CDK2 compromises host intrinsic antiviral immunity by phosphorylating and inactivating antiviral restriction factors such as SAMHD1, thereby facilitating the replication of viruses, including HIV-1 [75]. GSEA analysis revealed that high CDK2 expression is enriched in antiviral pathways such as the cytosolic DNA-sensing pathway, RIG-I-like receptor signaling, and viral life cycle pathways including hepatitis C and measles, suggesting a strong association with viral recognition and response mechanisms. Immune infiltration analysis further demonstrated a positive correlation between CDK2 and immunosuppressive or naïve immune cells (Tregs, naive CD4+ T cells, early-activated dendritic cells), alongside a negative correlation with effector immune subsets, such as activated memory CD4+ T cells and follicular helper T cells. This immune profile implies that elevated CDK2 expression may promote an immunosuppressive microenvironment conducive to viral persistence [76,77,78].

These findings suggest that the pharmacological modulation of MAPK3 and CDK2 may represent a viable strategy for restoring antiviral immunity and inhibiting viral propagation. Although testosterone has been implicated in immune regulation, direct evidence linking testosterone to the regulation of MAPK3 or CDK2 remains limited. Considering that testosterone can influence inflammation and immune cell composition via AR signaling, it is plausible that it may indirectly modulate MAPK3 and CDK2 activity through pathways such as AR/PTEN/PI3K/Akt. However, the specific mechanisms by which testosterone affects these kinases during viral infection require further mechanistic investigation.

## 4. Materials and Methods

### 4.1. Identification of Active Compounds and Corresponding Targets

The active compounds of *Moschus* were identified through comprehensive literature mining and HERB database (http://herb.ac.cn/) [accessed on 14 March 2025] retrieval. Compounds without known targets, with overly complex structures, or lacking pharmacological relevance were excluded. The remaining compounds were further screened based on pharmacokinetic and drug-likeness criteria.

For compounds available in the TCMSP database (https://old.tcmsp-e.com/tcmsp.php) [accessed on 14 March 2025], oral bioavailability (OB) ≥ 30% and drug-likeness (DL) ≥ 0.18 were set as thresholds. Compounds not available in TCMSP were searched in the PubChem database (https://pubchem.ncbi.nlm.nih.gov/) [accessed on 14 March 2025] to obtain their Simplified Molecular Input Line Entry System (SMILES) structures. These compounds were then input into the SwissADME platform (http://www.swissadme.ch/) [accessed on 14 March 2025] to predict their drug-likeness, and filtered according to Lipinski’s Rule of Five (molecular weight ≤ 500 Da, miLogP ≤ 5, number of hydrogen bond donors ≤ 5, number of hydrogen bond acceptors ≤ 10).

### 4.2. Retrieval of Genes Associated with Viral Respiratory Tract Infections

Disease-related targets associated with “viral respiratory tract infections” were retrieved from four publicly available databases: the Therapeutic Target Database (TTD) (https://db.idrblab.net/ttd/) [accessed on 15 March 2025], GeneCards (https://www.genecards.org/) [accessed on 15 March 2025], OMIM (https://omim.org) [accessed on 15 March 2025], and PharmGKB (https://www.pharmgkb.org/) [accessed on 15 March 2025]. The collected targets were merged and deduplicated to generate a non-redundant list of genes associated with VRTIs.

### 4.3. Construction of the Protein–Protein Interaction (PPI) Network and Screening of Core Targets

The overlapping targets between the predicted active compound targets and disease-related targets were identified as potential key targets for therapeutic intervention and used for PPI network construction. These targets were imported into the STRING database (https://cn.string-db.org/) [accessed on 17 March 2025] for PPI analysis, with a confidence score threshold of 0.4. Disconnected nodes were removed, and the resulting interaction data were visualized and analyzed using Cytoscape 3.10.3.

To identify core targets, the CytoNCA plugin in Cytoscape was employed to calculate six topological parameters: Betweenness Centrality (BC), Closeness Centrality (CC), Degree Centrality (DC), Eigenvector Centrality (EC), Local Average Connectivity (LAC), and Network Centrality (NC) [79]. The median value of each parameter was calculated. Further screening was conducted by progressively applying the median of the retained targets’ values to narrow down the range, ultimately identifying the most essential hub targets in the network.

### 4.4. GEO-Based Differential Gene Expression Analysis

Gene expression datasets related to VRTIs were obtained from the Gene Expression Omnibus database (GEO, https://www.ncbi.nlm.nih.gov/geo/) [accessed on 21 March 2025] by searching the keyword “viral respiratory tract infections”, with the organism set to Homo sapiens and the data type specified as “expression profiling by array.” Data preprocessing and analysis were conducted in R software (version 4.2.3). Multiple datasets were integrated using the inSilicoMerging package, and batch effects were corrected using the method described by Johnson et al. [80]. Differentially expressed genes (DEGs) were identified from the corrected expression matrix using the limma package, with thresholds set at |logFC| > 2 and *p* < 0.05.

### 4.5. Weighted Gene Co-Expression Network Analysis

To systematically identify gene co-expression modules associated with infection status, Weighted Gene Co-expression Network Analysis (WGCNA) was applied to the expression matrix for network construction and module detection. Raw expression data were first standardized, and genes with low variance (standard deviation < 0.5) were excluded to enhance network robustness. Hierarchical clustering of samples was performed to identify and remove outliers. The pickSoftThreshold function was used to determine the optimal soft-thresholding power, enabling the construction of an adjacency matrix and a topological overlap matrix (TOM) consistent with scale-free network topology. Genes were then clustered based on TOM dissimilarity, and initial modules were identified using the dynamic tree cut algorithm with a minimum module size of 50 genes. Module eigengenes (MEs) were calculated, and modules with highly correlated eigengenes (correlation ≥ 0.75) were merged. Finally, Pearson correlation analysis was performed between module eigengenes and infection phenotypes to identify modules significantly associated with infection status.

### 4.6. Enrichment Analysis

To explore the biological functions and pathways associated with key genes, Gene Ontology (GO) and Kyoto Encyclopedia of Genes and Genomes (KEGG) enrichment analyses were conducted using the clusterProfiler package in R. Gene symbols were converted to Entrez IDs using the org.Hs.eg.db database. GO analysis covered three categories: Biological Process, Cellular Component, and Molecular Function, with thresholds set at *p* < 0.05 and q < 1. the Top 10 enriched terms ranked by significance were selected for display. KEGG pathway enrichment analysis was conducted using the same threshold criteria. Enrichment results were visualized using circlize-based circular plots.

To further investigate functional pathways under different expression states, Gene Set Enrichment Analysis (GSEA) was performed. Samples were divided into high-expression and low-expression groups using the median expression value as the cutoff. A linear model was constructed, and differential expression analysis was performed across all genes using the limma package. Genes were ranked by log2 fold change, and GSEA was carried out using gseKEGG, with *p* < 0.05 as the cutoff and Homo sapiens as the species. Significantly enriched pathways were visualized using heatmaps, volcano plots, and enrichment curves. Top pathways were selected for further functional annotation to elucidate the biological relevance of key genes.

### 4.7. Immune Cell Infiltration Analysis

The relative abundances of 22 immune cell types in each sample were estimated using the CIBERSORT algorithm, based on batch-effect-corrected expression data, with the LM22 signature matrix and 100 permutations. Immune cell types with zero abundance across all samples were excluded. Samples were grouped by experimental condition, and immune infiltration patterns were visualized using stacked bar plots and violin–box plots generated with ggplot2. Statistical differences between groups were assessed using the Wilcoxon rank-sum test, with *p* < 0.05 considered significant.

To further investigate the relationship between differentially expressed core genes and immune cell infiltration, Spearman correlation analysis was performed between normalized gene expression levels and immune cell proportions. Correlation coefficients and *p*-values were calculated, and significant associations with *p* < 0.05 were visualized using lollipop plots.

### 4.8. Molecular Docking Validation

Core target proteins were selected as molecular docking targets. Protein structures were obtained from the UniProt and RCSB PDB databases (https://www.rcsb.org/) [accessed on 14 April 2025]. Structures were filtered based on resolution (<2.5 Å), experimental method (X-ray crystallography), species (Homo sapiens), chain length, and the presence of bound ligands. Downloaded crystal structures were processed using PyMOL (version 3.1.3) software to remove water molecules and small ligands. Receptors were prepared in AutoDockTools (version 1.5.7) by adding non-polar hydrogens, calculating charges, ensuring rigid receptor conformation, and saving as pdbqt files. Small-molecule ligands, selected from the previously screened active compounds, were downloaded from the TCMSP database in mol2 format or from PubChem in SDF format. All ligands were converted to the appropriate format using OpenBabel software (version 3.1.1). Ligand structures were further refined in PyMOL by removing water molecules and co-crystallized small ligands. Grid box parameters for molecular docking were defined using AutoGrid. The dimensions of the grid box were set to sufficiently encompass the entire binding site of the receptor. Grid spacing was adjusted within the permissible range of 0.2–1.0 Å to ensure comprehensive coverage of the binding pocket and to accommodate all plausible ligand conformations during docking. Molecular docking was performed in AutoDock to calculate binding energies, and results were visualized with PyMOL to illustrate molecular interactions. In this study, the term “receptor” refers exclusively to the macromolecular target proteins involved in docking simulations, regardless of their classical biological receptor functions.

## 5. Conclusions

This study systematically elucidated the potential mechanisms of *Moschus* in treating VRTIs using network pharmacology, transcriptomic analysis, immune cell infiltration analysis, and molecular docking. The results highlight MAPK3, CDK2, and MCL1 as key targets that may be directly engaged by *Moschus*’s active compounds, thereby interfering with viral replication and modulating host immune responses. Among them, testosterone exhibited particularly strong binding affinities, positioning it as a promising candidate for further experimental validation. However, this study is limited by its reliance on in silico predictions without experimental validation, and the specific regulatory roles of testosterone regarding key targets remain to be confirmed. Future studies should incorporate in vitro and in vivo experiments to validate the efficacy and mechanisms of *Moschus* and its compounds against VRTIs. These findings not only provide a systematic view of the mechanisms of action of *Moschus* but also offer a promising framework for guiding future drug discovery and the development of novel therapeutic interventions against VRTIs.

## Figures and Tables

**Table 1:** Information about selected active compounds of *Moschus*.

ID	Compound	CID	Structure
sx1	cyclopentadec-4-en-1-one	6365389	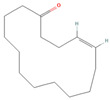
sx2	muscone	10947	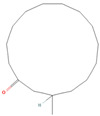
sx3	4-Cholesten-3-one	91477	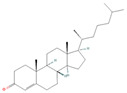
sx4	etiocholanedione	440114	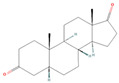
sx5	testosterone	6013	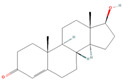
sx6	Androst-4-ene-3,17-dione	6128	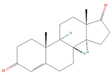
sx7	Androsta-4, 6-diene-3,17-dione	12452	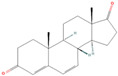
sx8	cyclotetradecanone	77153	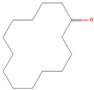
sx9	Estradiol	5757	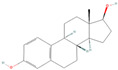
sx10	Morin	5281670	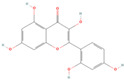
sx11	Muscopyridine	193306	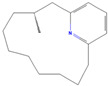
sx12	N-Nornuciferine	12313579	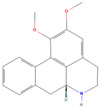

**Table 2:** Topological parameters of the 21 core genes identified by CytoNCA.

Gene	Degree	Eigenvector	LAC	Betweenness	Closeness	Network
EGFR	92	0.26643398	27.739130	549.591200	0.74468080	70.074580
ESR1	84	0.25030932	27.238094	412.316000	0.71428573	61.156887
SRC	82	0.25210682	29.073172	349.617130	0.70000000	64.178680
MMP9	76	0.22851463	25.789474	276.686520	0.67961160	53.860302
PTGS2	72	0.20763281	23.777779	315.760860	0.67307690	48.752533
MAPK3	66	0.21045792	24.242424	178.203340	0.64814810	42.785760
GSK3B	62	0.20833590	25.806452	143.282380	0.64220184	40.097504
PARP1	56	0.18899340	24.142857	156.978240	0.61403507	36.787210
PIK3R1	50	0.17424053	24.320000	48.420315	0.57851240	33.480170
ABCB1	50	0.15592830	19.040000	248.565280	0.60344830	29.815170
KDR	48	0.17763872	25.00000	48.865566	0.58333330	31.465607
MMP2	48	0.16904768	22.666666	77.597950	0.58823530	29.767578
MCL1	46	0.17670754	25.391304	45.075626	0.57851240	30.130154
IGF1R	44	0.16818203	24.181818	38.544117	0.58333330	28.329945
NR3C1	42	0.14626466	19.619047	51.647660	0.57377046	24.440815
CDK1	38	0.13185613	18.526316	54.070980	0.55555560	22.845032
PPARA	38	0.12817337	16.631578	94.413310	0.56000000	21.011786
CDK2	36	0.12666881	18.222221	81.468500	0.55118110	21.162943
TERT	36	0.13536796	19.555555	37.084934	0.5600000	21.636280
MAPT	34	0.11174154	14.117647	66.179740	0.5511811	16.139435
TH	32	0.09589354	15.000000	37.243458	0.5426357	18.194157

**Table 3:** Characteristics of selected GEO datasets.

GSE Accession	Participants	Samples	Platform
GSE38900	8 controls; 28 infected cases	Whole blood	GPL10558 (Illumina HumanHT-12 V4.0)
GSE63990	86 controls; 113 infected cases	Whole blood	GPL571 (Affymetrix Human Genome U133A 2.0)
GSE53545	102 controls; 94 infected cases	PBMCs	GPL10558 (Illumina HumanHT-12 V4.0)

## Data Availability

Data is contained in the paper.

## Abbreviations

The following abbreviations are used in this manuscript:

VRTIs	Viral respiratory tract infections
SMILES	Simplified Molecular Input Line Entry System
PPI	Protein–protein interaction
BC	Betweenness Centrality
CC	Closeness Centrality
DC	Degree Centrality
EC	Eigenvector Centrality
LAC	Local Average Connectivity-based method
NC	Network Centrality
GEO	Gene Expression Omnibus
DEGs	Differentially expressed genes
WGCNA	Weighted gene co-expression network analysis
PBMCs	Peripheral blood mononuclear cells
GSEA	Gene Set Enrichment Analysis
Tregs	Regulatory T cells
MAPK3	Mitogen-activated protein kinase 3
ERK1	Extracellular signal-regulated kinase 1
AR	Androgen receptor
